# Synergistic Effect of He for the Fabrication of Ne and Ar Gas-Charged Silicon Thin Films as Solid Targets for Spectroscopic Studies

**DOI:** 10.3390/nano14080727

**Published:** 2024-04-21

**Authors:** Asunción Fernández, Vanda Godinho, José Ávila, M. Carmen Jiménez de Haro, Dirk Hufschmidt, Jennifer López-Viejobueno, G. Eduardo Almanza-Vergara, F. Javier Ferrer, Julien L. Colaux, Stephane Lucas, M. Carmen Asensio

**Affiliations:** 1 Instituto de Ciencia de Materiales de Sevilla (CSIC-Univ. Seville), Avda. Américo Vespucio 49, 41092 Seville, Spain; godinho@icmse.csic.es (V.G.); cjimenez@icmse.csic.es (M.C.J.d.H.); dirk@icmse.csic.es (D.H.); jennilop@ucm.es (J.L.-V.); gealmanzav@unal.edu.co (G.E.A.-V.); 2 Synchrotron SOLEIL, Universite Paris-Saclay, L’ Orme des Merisiers, BP48, 91190 Saint-Aubin, France; jose.avila@synchrotron-soleil.fr (J.Á.); mc.asensio@csic.es (M.C.A.); 3 Centro Nacional de Aceleradores (Univ. Seville, J. Andalucía, CSIC), Av. Tomas Alva Edison 7, 41092 Seville, Spain; fjferrer@us.es; 4 Departamento de FAMN (Univ. Seville), Aptd. 1065, 41012 Seville, Spain; 5 Laboratoire d’Analyse par Réactions Nucléaires (LARN), Namur Institute of Structured Matter (NISM), University of Namur, 61 Rue de Bruxeles, 5000 Namur, Belgium; julien.colaux@unamur.be (J.L.C.); stephane.lucas@unamur.be (S.L.); 6 Madrid Institute of Materials Science (ICMM), CSIC, Cantoblanco, 28049 Madrid, Spain

**Keywords:** magnetron sputtering, gas-charged Si films, microstructural characterization, IBA analysis, XPS and XAS spectroscopic analyses, Ne, Ar and He solid targets

## Abstract

Sputtering of silicon in a He magnetron discharge (MS) has been reported as a bottom-up procedure to obtain He-charged silicon films (i.e., He nanobubbles encapsulated in a silicon matrix). The incorporation of heavier noble gases is demonstrated in this work with a synergistic effect, producing increased Ne and Ar incorporations when using He–Ne and He–Ar gas mixtures in the MS process. Microstructural and chemical characterizations are reported using ion beam analysis (IBA) and scanning and transmission electron microscopies (SEM and TEM). In addition to gas incorporation, He promotes the formation of larger nanobubbles. In the case of Ne, high-resolution X-ray photoelectron and absorption spectroscopies (XPS and XAS) are reported, with remarkable dependence of the Ne 1s photoemission and the Ne K-edge absorption on the nanobubble’s size and composition. The gas (He, Ne and Ar)-charged thin films are proposed as “solid” targets for the characterization of spectroscopic properties of noble gases in a confined state without the need for cryogenics or high-pressure anvils devices. Also, their use as targets for nuclear reaction studies is foreseen.

## 1. Introduction

The dominating feature of inert gas atoms implanted in most solids via ion beam irradiation over a wide energy range (500 keV–100 eV) is their high heat of solution, leading to an essentially zero solubility and gas-atom precipitation (formation of small “bubbles”) [1,2,3,4,5,6]. He has been particularly investigated due to its technological interest in studying damage in nuclear reactor materials [7,8]. The implantation of other noble gases such as Ne, Ar and Xe has also been investigated [9,10,11], showing the accumulation of gas trapped in bubbles. Implantation studies refer to a “top-down” methodology with interest in studying materials’ degradation in nuclear reactors [7,8] and defect engineering in electronic device development [12,13]. More recently, several works have investigated the “bottom-up” magnetron sputtering (MS) deposition in He plasmas, leading to the tailored fabrication of nanostructured carbon films [14], nanoporous Al [15] or He-charged films [16,17].

In particular, films fabricated via MS with the formation of nanopores or nanobubbles (He-filled nanopores) are attracting growing interest in new materials and applications [17,18,19,20,21,22,23]. These include, among others, optical devices [19], electrodes in batteries [20,21] or catalysts [23]. An exhaustive microstructural characterization also showed that gas content and nanobubble size and shape are finely tunable [17,24,25]. Due to the high density and pressure of He trapped in the nanobubbles [24,25,26,27], the He-charged Si films have been proposed as “solid targets” for nuclear reaction studies in our previous works [28,29,30,31,32,33].

Building on this background, the first goal of the present work was to evaluate the incorporation of heavier noble gases such as Ar and Ne during the MS deposition of silicon films. The pure Ar MS process has been widely investigated due to its relatively high abundance and high sputtering yield [34]. Ar incorporation has been reported for different film compositions, showing a modification of mechanical or electrical properties [35,36] and formation of bubbles [37]. Previous works also investigated the use of Ar–He gas mixtures for the deposition of Ti [16,18] and C [14], in which Ar partial pressure enabled a high sputtering rate to be maintained. In reference [15], Al films were also deposited with different Ar–He gas mixtures. However, only He incorporation was reported in these previous works. In another work [38], including a growth model, we report that the chemical nature of the sputter gas affects not only the sputtering mechanism of the Si target but also the film growth mechanism. In particular, He introduces a degree of mobility, resulting in the coarsening of small pores [38]. The main results of the use of Ne and Ne–He mixtures in MS deposition relate to film deposition rates and properties [39,40,41]. Controlled incorporation of Ne and Ar has also been reported during High Power Impulse MS deposition in Ar–Ne gas mixtures [42]. Investigated matrix materials included vanadium [39], carbon [40,41] and tungsten [42].

The work presented in this article builds upon previous knowledge and aims to increase the Ne and Ar content in gas-charged silicon films. A synergistic effect is demonstrated, showing that adding He to the plasma gas mixture effectively promotes the incorporation of Ne and Ar into the nanobubbles. In addition to microstructural (SEM and TEM) and elemental composition (IBA) characterizations, XAS and XPS spectroscopic studies were undertaken for Ne-charged Si films synthesized via MS with Ne and He–Ne gas mixtures. Our results were analyzed for the Ne K-edge excitation and 1s binding energies and discussed considering data previously reported for Ne and He bubbles obtained through ion implantation in aluminum [43,44] or martensitic steel [45]. Previous spectroscopic data were also obtained under conditions of cryo-condensation for He and Ne [44,46] or high-pressure devices for He [47]. New results for the gas-charged films fabricated via MS are presented and discussed here.

Based on the results presented in this article, the investigated Si films are thought to be of interest for the study of spectroscopic properties of condensed noble gases without the need for cryogenic, high-pressure anvils or ion implanter devices. The fabrication of these Ne and Ar solid targets is also of interest for nuclear reaction studies [28,29,30,31,32,33].

## 2. Materials and Methods

### 2.1. Film Preparation

Si films were prepared in a magnetron sputtering (MS) deposition chamber (residual vacuum in the range 1 × 10^−6^ mbar) operated with one magnetron head furnished with a 2-inch Si cathode placed at 30° towards the sample holder. In our earlier work [17], we demonstrated that He-filled nanopores are formed both for a cathode placed on top (parallel to the substrate holder) or for tilted deposition geometries. The selected tilted geometry in this work was maintained for all samples prepared, aiming for a comparative study of the synergistic effects reported here. The Si target was supplied by Neyco with 99.999 % purity. The distance from the target to the substrate was 5 cm vertically, and the sample holder was rotated during deposition. In the Supporting Information File S1, a schematic drawing of the experimental set-up has been included (Figure S1). As process gas, we used He, Ne and Ar supplied by Air Liquid with 99.999% purity. Table 1 summarizes the nomenclature of the investigated samples along with their deposition parameters (gas pressures, power and time) and deposition rate (derived from film thickness measurements). Thin films were grown on 100 Si wafer substrates (0.5 mm thick) using a magnetron from the AJA (Scituate, MA, USA) Company with an unbalanced magnetic field configuration. For operation, power supplies from Cesar RF-Dressler and Advance Energy-Pinnacle Plus were respectively used in RF and DC mode with constant power. The sample holder was at a floating potential and was not cooled during the process. 

### 2.2. Films Characterization (Microstructure and Elemental Composition)

The thickness and morphology of the films were examined using scanning electron microscopy (SEM) employing a HITACHI S-4800 SEM-FEG microscope (Tokyo, Japan) operated at 1−2 kV. The samples deposited on silicon substrates were cleaved for cross-sectional views. The nanostructure of the nanocomposite films was investigated at the Laboratory of Nanoscopies and Spectroscopies (LANE-ICMS, Sevilla, Spain) via transmission electron microscopy (TEM) using a JEOL 2100Plus (Tokyo, Japan) and a FEI-Tecnai G2 F30 TEM (Eindhoven, Netherlands) operated at 200 and 300 kV, respectively. The cross-sectional TEM lamellas were prepared through mechanical polishing and dimple grinding of the coatings deposited on silicon, followed by Ar^+^ ion milling to achieve electron transparency. Representative porous areas were selected for imaging and analysis. The pore distribution was evaluated from TEM micrographs by binarizing them and using the “Analyze Particle” function of ImageJ software (version 1.50b) [48].

Si, Ne, Ar and He content were derived from Ion Beam Analysis (IBA) carried out at the National Centre for Accelerators (CNA, Seville, Spain) using a 2.0 MeV proton beam and a passivated implanted planar-silicon (PIPS) detector set at 165°. Data analyses were performed via simulations with the SIMNRA code [49]. For the case of S1 and S2 samples, a complete IBA analysis, including possible impurities (C, O and H), was additionally carried out at the SIAM platform of the University of Namur (Belgium) using a 2M-Tandetron Linear Accelerator from HVEE (Amersfoort, Netherlands). The following conditions were used: (i) With the alpha beam, the samples were analyzed at 2.4 MeV in tilted incidence to determine the H content through ERD (elastic recoil detection). Then, EBS (elastic backscattering spectrometry) spectra were collected from the same location at various incident energies, namely, at 3.05 MeV to determine the oxygen content [50], at 3.75 MeV to determine the nitrogen content [51], and at 4.3 MeV to determine the carbon content [52]. (ii) The samples were then analyzed with a proton beam at 1.96 MeV (p-EBS) for sensitivity to Ne and He. The set of 5 spectra acquired on each sample was self-consistently fitted with DataFurnace [53], using the stopping power provided by the SRIM database (www.srim.org)) as well as the evaluated cross-section functions available on the SigmaCalc [54], for extracting the elemental depth profiles.

### 2.3. Films Characterization (Spectroscopic Studies)

X-ray photoelectron spectroscopy (XPS) spectra were recorded with a SPECS electron spectrometer (Berlin, Germany) equipped with a PHOIBOS 150 hemispherical analyzer (Berlin, Germany) using Al Kα radiation with a 35 eV pass energy and normal emission take-off angle. This configuration gives an energy resolution of <0.4 eV. The Si films deposited on silicon wafer pieces were analyzed as received and after a gentle Ar sputtering (2.70 keV). The spectra were calibrated with the Si 2p signal at 99.2 eV.

X-ray absorption spectroscopy (XAS) at the Ne K-edge was carried out at the beamline Antares of the Synchrotron Soleil in France. Further details can be found in Ref. [55]. Spectra were measured in the 855 to 900 eV photon range by fluorescence emission yields using a fluorescence detector from Bruker Karlsruhe, Germany). All spectra were normalized using the incoming flux, measured from a thin grid with freshly evaporated gold, placed upstream of the sample chamber with a relative energy precision of ±25 meV in the energy range for the Ne K-edge. The XAS spectra were referenced to the carbon dip, which has been calibrated to 284.7 eV using HOPG. Data were also normalized to a linear background function at higher energy. 

## 3. Results and Discussion

### 3.1. Selection of Experimental Conditions for the Fabrication of Investigated Gas-Charged Silicon Films

Deposition conditions were selected with the aim of elucidating the effect of He incorporation in MS deposition of Si films when using Ne (or Ar) as process gas. Table 1 summarizes three selected cases of study for the case of Ne: (i) Samples S1, S2 and S3 grown at 150 W in dc mode using pure Ne (S1) and two different Ne–He mixtures (S2 and S3); (ii) Samples S4 and S5 grown at 150 W in rf mode using pure Ne (S4) and one Ne–He mixture (S5). (iii) Samples S6 and S7 grown at 300 W in dc using pure Ne (S6) and one Ne–He mixture (S7). For the case of Ar, two samples were fabricated at 150 W in dc mode using pure Ar (S8) and one Ar + He mixture (S9). In the present work, the main investigated operation mode is dc. Samples S4 and S5 have also been included to demonstrate that, in rf mode, a synergistic effect occurs for the Ne incorporation. In addition, the comparison of samples S1 and S4 provides data to compare the effect of using dc or rf mode on the Ne incorporation for a pure Ne plasma.

### 3.2. The Microstructure and Elemental Composition of Ne-Charged Silicon Thin Films

**Figure 1:**
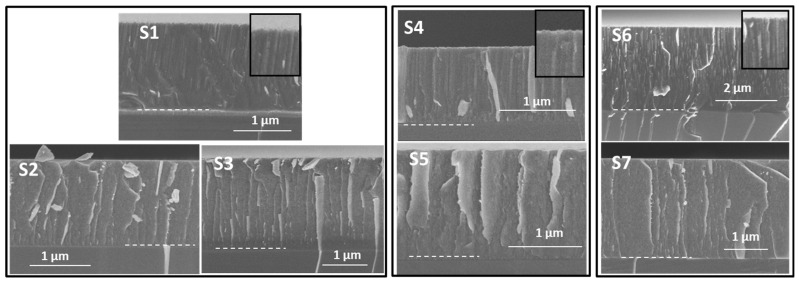
Cross-section SEM images of samples 1 to 7. Samples 1, 4 and 6 correspond to Si-Ne. Samples 2, 3, 5 and 7 correspond to Si-Ne(He). Notes: (i) Scales are not equal to visualize entire layers maximizing magnification. (ii) For the Si-Ne samples zoom images show columnar microstructure. (iii) Dash lines mark the interface between the silicon substrate and the coating.

**Figure 2:**
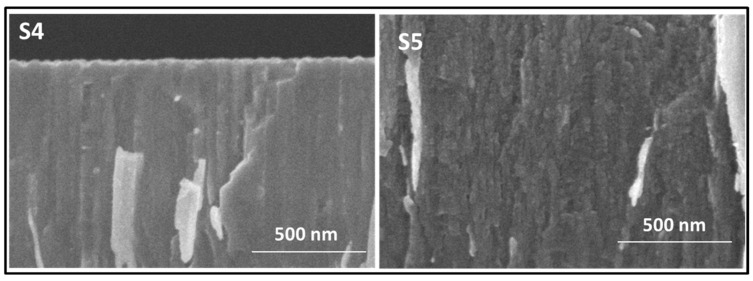
Cross-section SEM images at high magnification for representative S4 (Ne) and S5 (Ne + He) samples.

Figure 1 presents an overview of cross-section SEM images obtained for the samples fabricated to investigate the Ne incorporation (samples 1 to 7 in Table 1). The images are grouped considering the three cases of study described above. The thickness of the Si films was determined from the cross-section SEM images and is included in Table 2. A columnar structure is observed in the zoomed images at the top border of the cross-section for samples grown in pure Ne (S1, S4 and S6). Higher magnification SEM images for representative samples S4 and S5 are presented in Figure 2. The Si-Ne film (S4) shows a dense columnar microstructure, while the Si-Ne(He) film (S5) shows the presence of nano porosity/nanobubbles. Silicon films fabricated through MS with Ne and Ne–He have proved to be amorphous via X-ray diffraction, as we previously found for the He-charged films [25]. Please refer to Figure S2 in the Supporting Information for additional details.

**Figure 3:**
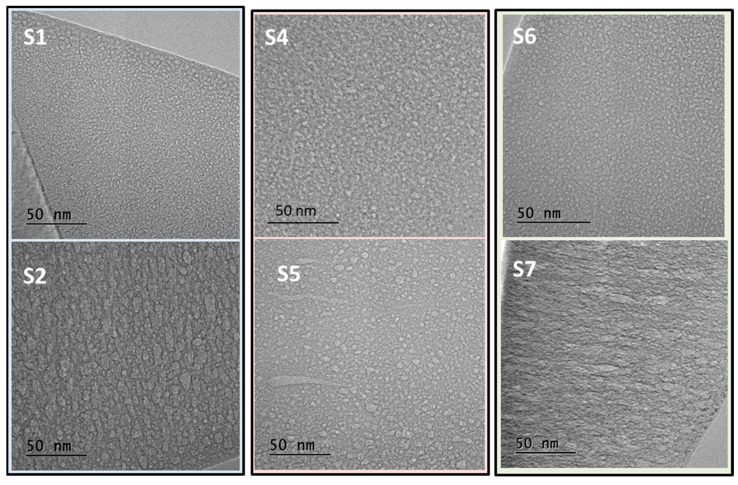
TEM cross-section images for representative samples: 1, 4 and 6 correspond to Si-Ne; 2, 5 and 7 correspond to Si-Ne(He).

For a further higher magnification study, images from cross-section TEM lamellas are presented in Figure 3. Samples grown in pure Ne show the formation of small nanopores. The addition of He to the deposition process leads to the formation of bigger nanopores with broad size and aspect ratio distributions. Data analysis and corresponding histograms are presented in Supporting Information (Figures S3–S5) and summarized in Table 2. Note that the pore size is defined as the diameter of a circle equivalent in area to the one obtained for each pore in the TEM images. This allows for the comparison of pores with different elongated shapes. Additionally, the cross-section TEM analysis shows in some images the characteristic intercolumnar porosity for films S1 and S4 grown in pure Ne. These images are included in the Supporting Information (Figure S6). Table 2 also shows that for pure Ne plasma, the dc mode produces larger pore sizes than the rf one. This result is in agreement with previous data for He-charged Si films in reference [25]. In addition, larger bubbles may be associated with lower gas densities, also according to previous results with He [24,26]. See also data in Table 3.

The TEM study showed numerous nano-pores (nano-voids) which may produce stress by deformation of the amorphous silicon matrix. For the charged films we have reported [24,26] a relationship between the surface tension of the nanopores and the pressure of the trapped gas; therefore smaller pores have typically higher gas densities. Although films are fragile and can break by stress, they have shown to be stable for years under a gentle manipulation.

**Figure 4:**
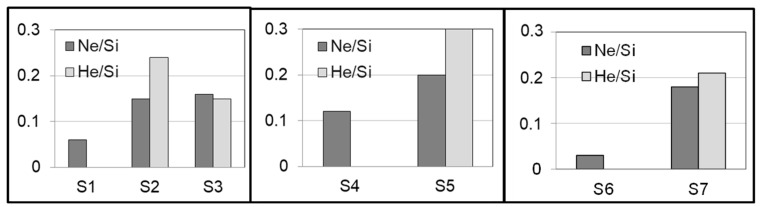
Bar diagram plots of atomic content ratio for the Si-Ne and Si-Ne(He) samples. For uncertainty values refer to Table 3.

**Figure 5:**
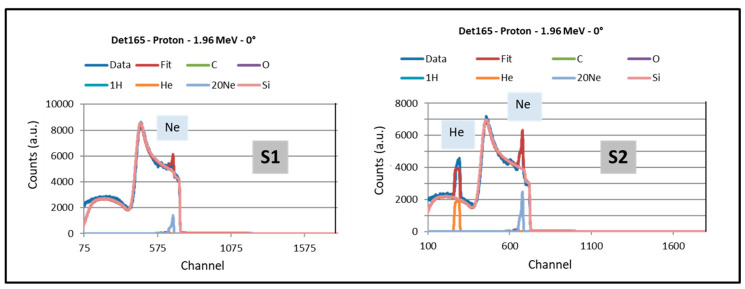
Proton beam EBS-spectra for representative samples: S1 (Ne) and S2 (Ne + He).

Film compositions were derived from IBA and are summarized in Table 3 and Table 4. In this work, an important advantage of this technique is the availability to quantify light elements such as He and possible H, C and O contaminants. Impurities typically come from residual vacuum species activated during MS deposition [25]. Representative impurities are reported in Table 4 for samples S1 and S2. Figure 4 shows bar diagrams of the Ne/Si and He/Si atomic ratios obtained for the investigated samples associated with the selected cases of study. The addition of He promotes the incorporation of Ne (and He) into the films together with the formation of numerous bigger and elongated nanopores, as described above from the microstructural analysis. To illustrate this synergistic effect of He for Ne incorporation, Figure 5 presents the p-EBS spectra obtained for samples S1 and S2. The peaks due to scattering with He and Ne are clearly identified, evidencing the desired increase of Ne incorporation. The results in this section are, therefore, relevant in the context of applications where a high amount of specific trapped gases is needed.

### 3.3. The Microstructure and Elemental Composition of Ar-Charged Silicon Thin Films

**Figure 6:**
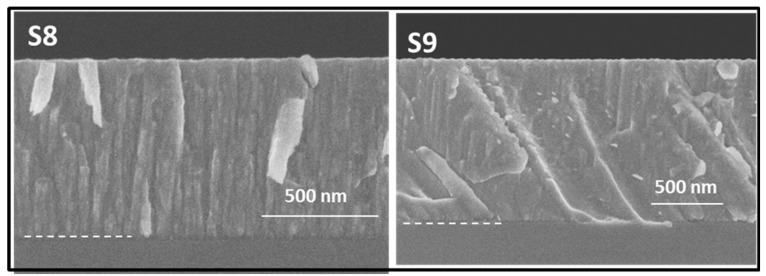
Cross-section SEM images for investigated S8 (Ar) and S9 (Ar + He) samples. Notes: (i) Scales are not equal to visualize entire layers maximizing magnification. (ii) Dash lines mark the interface between the silicon substrate and the coating.

**Figure 7:**
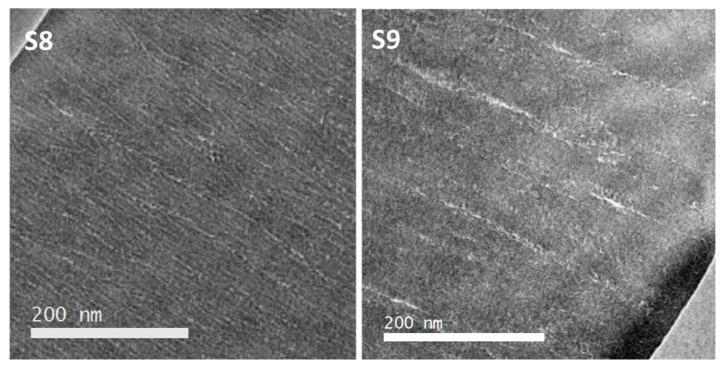
TEM cross-section images for the S8 (Ar) and S9 (Ar + He)) samples.

Figure 6 presents the cross-section SEM images obtained for the samples fabricated to investigate the Ar incorporation (samples 8 and 9 in Table 1). Both Si-Ar and Si-Ar(He) films show a columnar structure characteristic of Ar-assisted MS deposition. Thicknesses and column width range for the Si films were determined from these cross-section SEM images and are included in Table 2. Figure S6 in the Supporting Information includes additional top-view SEM images showing the columnar microstructures. Consistent data about column sizes were found from these top-view SEM images (see Figure S6). Note that due to the higher sputtering yield expected for Ar, shorter deposition times were used for samples S8 and S9. The prevalence of the columnar structure for the case of sample S9 is in agreement with the previously reported formation of Ar-dominated plasmas during MS deposition in Ar–He mixtures [15]. For a further higher magnification study, images from cross-section TEM lamellas are presented in Figure 7. Sample S8, grown in pure Ar, shows characteristic intercolumnar Ar trapped in defects, while the addition of He leads to the formation of larger intercolumnar gas accumulation. Data analysis regarding pore size and shape was not possible for these samples, although an increase in intercolumnar porosity is clearly observed.

**Figure 8:**
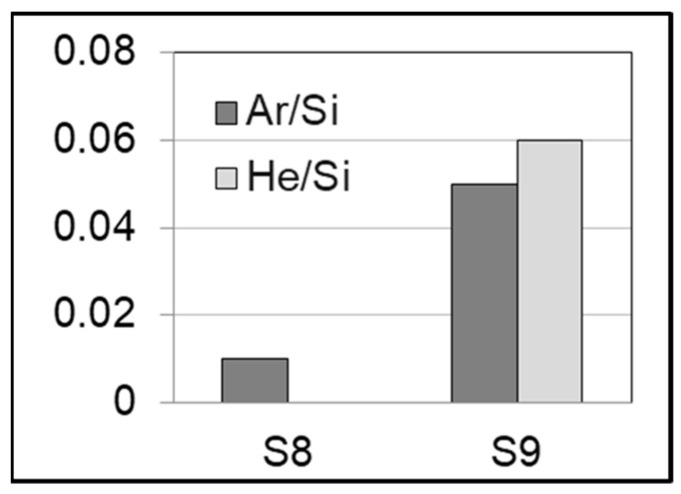
Bar diagram plots of atomic content ratios for the S8 (Ar) and S9 (Ar + He) samples. For uncertainty values refer to Table 3.

The film composition of the main elements was derived from IBA and is summarized in Table 3. Although the Ar-dominated plasma gave a similar columnar structure, and even if the amount of incorporated Ar is low (as compared to the Ne case), a synergistic effect was also found. Figure 8 shows bar diagrams of the obtained Ar/Si and He/Si atomic ratios for the two samples. The addition of He promotes the Ar (and He) incorporation together with larger intercolumnar nanoporosity, as described above from the microstructural analysis. Again, these results are relevant for applications where a high amount of specific trapped gases is needed.

### 3.4. Spectroscopic Study of the Ne1s Binding Energy of Ne-Charged Si Films by XPS

**Figure 9:**
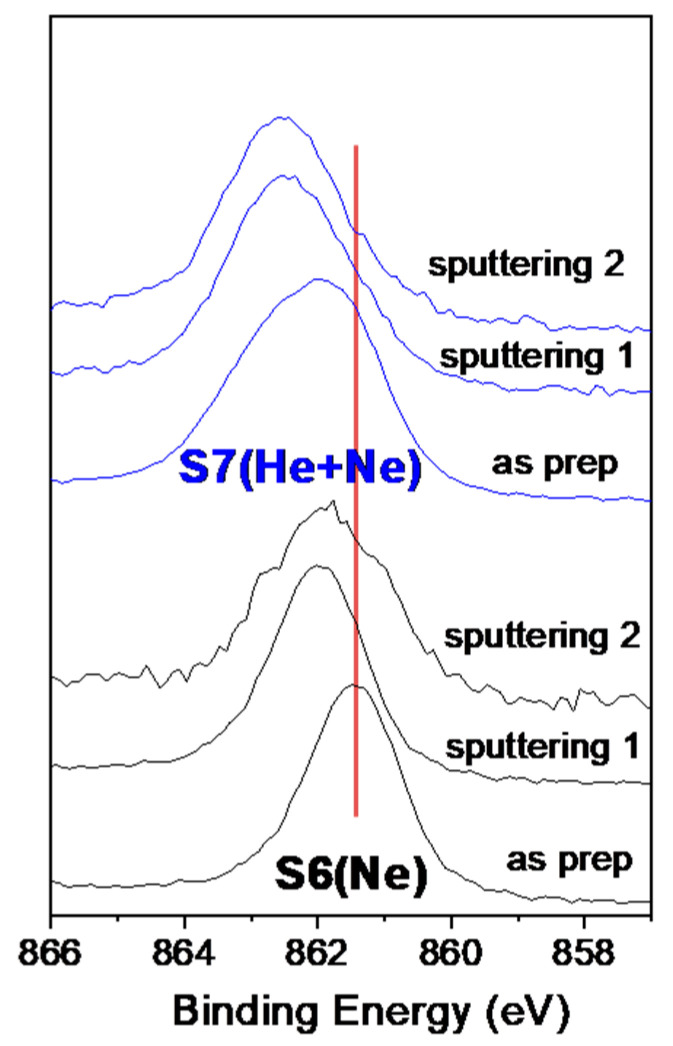
Normalized Ne1s XPS spectra for the as prepared amples S6 (Ne) and S7 (Ne + He). The spectra after successive Ar^+^ sputtering for 2 and 10 are also shown. The red line is a reference to visualize peaks shifts.

Ne in the gas phase has a binding energy (Eb) for the 1s level of around 870–866 eV. When trapped in bubbles, typically in a metallic matrix such as Al, it tends to shift to lower Eb [43]. The behavior is dominated by a final state effect associated with the screening of the photo-hole using the host-metal conduction electrons. The smaller the bubble, the greater the shift [43]. Similar results were also found for implanted Ar and Xe with respect to their gas phase Eb [10,56]. In this work, samples S6 and S7 have been selected for the XPS study of the Ne K edge binding energy. Figure 9 shows the Ne 1s photoelectron spectra for the as-prepared samples, showing the shift to a lower Eb (~861.5 eV) for sample S6 (grown in pure Ne) with smaller bubbles. The higher Ne 1s Eb (~862 eV) and the broader peak observed for the as-prepared S7 sample are also in agreement with the larger mean pore size and wider pore size distribution observed for this sample (5.2 ± 2.7 nm) vs. sample S6 (2.7 ± 0.8 nm). See Figure S4 in the Supporting Information. The increase in Ne 1s Eb after Ar sputtering in both samples may indicate that ion beam mixing effects could lead small bubbles to aggregate within larger bubbles.

### 3.5. Spectroscopic Study of the Ne-Kedge Absorption Spectra of Ne-Charged Si Films by XAS

Previous characterizations of the He K-edge via Vacuum Ultraviolet (VUV) absorption [57] and Electron Energy Loss spectroscopy (EELS) [58] already demonstrated an absorption energy increase (with respect to the gas) for He condensed or encapsulated in bubbles. This behavior for the He 1s→2p transition has been associated with the short-range Pauli pseudo-repulsion of the 2p-electron wave function with the ground-state orbital of neighboring atoms [59,60]. This effect has also been measured at the microscopic scale by monitoring individual bubbles in a transmission electron microscope using STEM–EELS [24,27,61]. For the case of gaseous Ne, the K-shell excitation has also been previously investigated through EELS using small-angle inelastic scattering of 2.5 keV electrons [62]. The relatively high amount of Ne incorporated in our Si films prompts us to consider the possibility of studying the Ne K-edge in the films. Due to the low cross-section that we observed for the STEM–EELS analysis, X-ray Absorption Spectroscopy (XAS) measurements were conducted at the synchrotron Soleil facilities and are presented here. Previous studies of the K-shell excitation with X-ray photons have also been reported for condensed Ne films [46].

**Figure 10:**
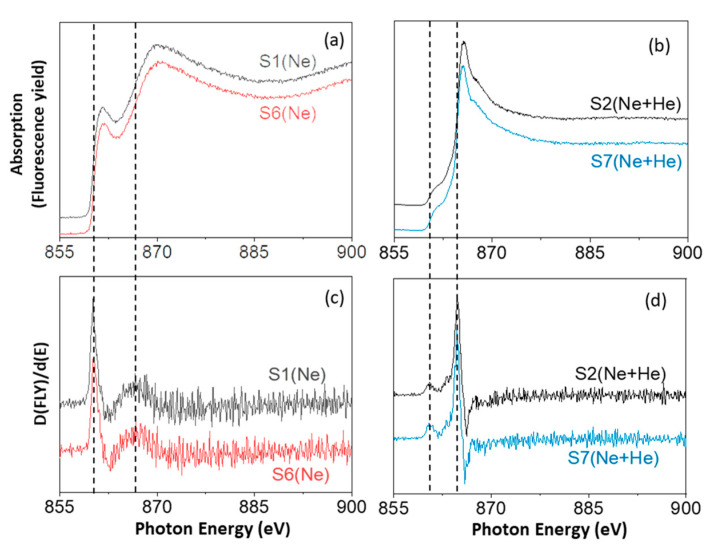
Normalized (**a**,**b**) and first derivative (**c**,**d**) of Ne 1s XAS spectra for samples S1 (Ne), S2 (Ne + He), S6 (Ne) and S7 (Ne + He).

In this work, samples S1–S2 and S6–S7 have been selected for the XAS study. The spectra in Figure 10 correspond to the Ne K-edge absorption for representative areas of the investigated samples. In addition to the spectra, the first derivative curves are included in Figure 10 to determine the inflection point (I.P.) of the absorption edges. According to EELS data for gaseous Ne reported in ref. [62], a first absorption peak (I.P. at 860.0–860.5 eV) was assigned to the optically forbidden 1s→3s electric quadrupole transition. It was followed by the allowed 1s→np absorption series (I.P. at 866.7 and 864.7 eV, respectively, for samples grown with Ne and Ne–He mixtures). In addition, results via X-ray photon absorption in ref. [46], using different probes and experimental techniques, allowed one to selectively access the electronically excited states at the surface and inside the bulk of condensed solid Ne films. This work demonstrated, using a surface-selective probe, that the dipole forbidden 1s→3s transition becomes allowed by admixture of 3pz character to the 3s orbital due to asymmetric squeezing in the reduced symmetry of the surface layer [46]. This surface effect explains our observation of relatively high intensity of the first absorption peak for samples (S1 and S6) grown in pure Ne. The gas is trapped in numerous small pores (see Figure 3 and Figure S3), maximizing the gas–matrix surface interaction at the pore borders. The second peak for the allowed 1s→np series shows a shift towards higher absorption energy for samples S1 and S6, as expected for condensed Ne in the smaller bubbles.

## 4. Conclusions and Perspectives

Previously, we have investigated the production of He-charged films via magnetron sputtering of silicon in pure He plasmas. Also, other authors have recently presented theoretical works describing He plasma sputtering deposition [63] and ion implantation methods [64]. In the present work, we show an improved fabrication of Ne and Ar-charged films when Ar–He or Ne–He gas mixtures are used (both in dc and rf mode). Ion Beam Analysis is a powerful tool to quantify the Ne, Ar and He contents within these solid–gas nanocomposite thin films. Microstructure and composition characterizations elucidated not only the amounts of trapped gases but also the effect of He addition on the size and shape distribution of bubbles associated with the trapped gases. Two effects can be concluded by adding He to the Ne or Ar MS deposition of silicon films: (i) He incorporation associated with increased Ne and Ar amounts; (ii) a larger mean pore size and a wider pore size distribution. For pure Ne plasma, the dc mode produces larger pore sizes and lower amounts of trapped Ne compared to the rf mode. The silicon films in this work have proved to be amorphous, as in the case of pure He [25]. In spite of the strong nanostructuration of the films (nanovoids/nanobubbles), the materials have shown to be stable for years under gentle manipulation.

For the case of Ne-charged silicon films, spectroscopic studies were conducted using XPS and XAS. The developed films are, therefore, of interest for characterizing the spectroscopic properties of noble gases trapped in bubbles in a condensed state without the need for cryogenic devices or high-pressure anvils. Effects on the Ne 1s binding energy and K-edge absorption energies, observed in XPS and XAS measurements, respectively, were related to final (screening of the photo-hole) and initial (Pauli repulsions) state effects correlated to the pore size distributions. The density and pressure of the gas within the bubbles are relevant factors to consider in future investigations. Our work using MS (bottom-up method) adds new knowledge to previous relevant works that have demonstrated the effect of ion irradiation (top-down method) on semiconductor electronic structure [65].

The results in this work are also important for applications of the “solid–gas” nanocomposite films as Ne (and Ar) solid targets for nuclear reaction experiments of interest in astrophysics and nuclear structure studies [28,29,32,33]. These solid targets can overcome the limitations of cryogenic or gas cell-based systems, which are bulky and difficult to handle, thus facilitating usage. As we have previously demonstrated for ^3^He [30,31], the methodology could also be extended in the future to the use of isotopes such as ^21^Ne. A methodology to reduce the gas consumption for film fabrication is available [30,31].

## Figures and Tables

**Table 1:** Nomenclature and deposition parameters for investigated samples.

Sample nr. Description	Deposition Time (h)	Sputtering Gas and Pressure (Pa)	Power (dc or rf) (W)	V (V)	I (A)	Deposition Rate * (nm/min)
S1: Si-Ne/150dc/2Ne	2	Ne(2)	150 (dc)	430	0.35	12.6 ± 0.4
S2: Si-Ne(He)/150dc/2Ne + 2He	2	Ne(2) + He(2)	150 (dc)	350	0.42	12.1 ± 0.3
S3: Si-Ne(He)/150dc/1Ne + 1He	3	Ne(1) + He(1)	150 (dc)	410	0.37	11.9 ± 0.3
S4: Si-Ne/150rf/2Ne	2	Ne(2)	150 (rf)	425 **		8.6 ± 0.2
S5: Si-Ne(He)/150rf/2Ne + 2He	3.5	Ne(2) + He(2)	150 (rf)	310 **		7.0 ± 0.1
S6: Si-Ne/300dc/2Ne	1.5	Ne(2)	300 (dc)	450	0.65	31.5 ± 0.5
S7: Si-Ne(He)/300dc/2Ne + 2He	1.5	Ne(2) + He(2)	300 (dc)	380	0.81	24.6 ± 0.4
S8: Si-Ar/150dc/2Ar	0.75	Ar(2)	150 (dc)	417	0.36	16.2 ± 0.5
S9: Si-Ar(He)/150dc/1Ar + 1He	1.25	Ar(1) + He(1)	150 (dc)	384	0.39	15.1 ± 0.3

* Calculated from deposition time and the thickness determined by SEM. ** DC-Bias.

**Table 2:** SEM and TEM microstructural analysis of investigated samples.

Sample nr. Description	Thickness (SEM) (µm)	Column Size Range (SEM) (nm)	Mean Column Size (SEM) (nm)	Pore Size Range (TEM) (nm)	Mean Pore Size (TEM) (nm)	Mean Aspect Ratio (TEM)
S1: Si-Ne/150dc/2Ne	1.51 ± 0.02			1–5	2.5 ± 0.9	0.6 ± 0.2
S2: Si-Ne(He)/150dc/2Ne + 2He	1.45 ± 0.01			1–12	5.5 ± 2.3	0.6 ± 0.2
S3: Si-Ne(He)/150dc/1Ne + 1He	2.14 ± 0.01					
S4: Si-Ne/150rf/2Ne	1.03 ± 0.01			1–5	1.7 ± 0.6	0.6 ± 0.2
S5: Si-Ne(He)/150rf/2Ne + 2He	1.47 ± 0.01			1–20	3.8 ± 1.8	0.6 ± 0.2
S6: Si-Ne/300dc/2Ne	2.84 ± 0.02			0.5–5	2.7 ± 0.8	0.7 ± 0.2
S7: Si-Ne(He)/300dc/2Ne + 2He	2.22 ± 0.01			1–14	5.2 ± 2.7	0.5 ± 0.2
S8: Si-Ar/150dc/2Ar	0.73 ± 0.01	20–90	38 ± 20			
S9: Si-Ar(He)/150dc/1Ar + 1He	1.13 ± 0.01	20–100	49 ± 26			

**Table 3:** Elemental composition for the investigated Si layers considering only main elements (Si, Ne, Ar and He).

Sample nr. Description	at% Si	at% Ne	at% Ar	at% He	Atomic Ratio Ne/Si or Ar/Si	Atomic Ratio He/Si
S1: Si-Ne/150dc/2Ne	94.1 ± 2.3	5.90 ± 0.25			0.063 ± 0.004	
S2: Si-Ne(He)/150dc/2Ne + 2He	71.8 ± 1.8	10.95 ± 0.50		17.24 ± 0.90	0.15 ± 0.01	0.24 ± 0.02
S3: Si-Ne(He)/150dc/1Ne + 1He	76.3 ± 2.3	12.10 ± 0.61		11.62 ± 0.58	0.16 ± 0.01	0.15 ± 0.01
S4: Si-Ne/150rf/2Ne	89.5 ± 2.7	10.5 ± 0.53			0.12 ± 0.01	
S5: Si-Ne(He)/150rf/2Ne + 2He	66.9 ± 2.0	13.0 ± 0.65		20.1 ± 1.0	0.19 ± 0.01	0.30 ± 0.02
S6: Si-Ne/300dc/2Ne	97.0 ± 2.9	3.0 ± 0.30			0.031 ± 0.004	
S7: Si-Ne(He)/300dc/2Ne + 2He	71.6 ± 2.1	13.0 ± 0.65		15.36 ± 0.77	0.18 ± 0.01	0.21 ± 0.02
S8: Si-Ar/150dc/2Ar	99 ± 1		1.00 ± 0.15		0.010 ± 0.001	
S9: Si-Ar(He)/150dc/1Ar + 1He	89 ± 10		4.8 ± 0.3	5.7 ± 0.5	0.05 ± 0.01	0.06 ± 0.01

**Table 4:** Elemental content for main contaminants in samples S1 and S2.

Sample nr. Description	at% C	at% O	at% H
S1: Si-Ne/150dc/2Ne	1.30 ± 0.07	1.55 ± 0.29	2.10 ± 0.11
S2: Si-Ne(He)/150dc/2Ne + 2He	1.22 ± 0.08	2.85 ± 0.38	2.74 ± 0.27

## Data Availability

The data presented in this study are available on request from the corresponding author.

## Supplementary Materials

The following supporting information can be downloaded at: https://www.mdpi.com/article/10.3390/nano14080727/s1. Figure S1: Schematic drawing of the magnetron sputtering experimental set-up, Figure S2: Representative X-ray diffractograms for samples S1, S2 and S4 deposited onto fussed quartz amorphous substrates, Figure S3: Study of samples S1 and S2. Pore (or trapped gas nanobubble) size and aspect ratio distribution histograms from TEM cross-sectional images, Figure S4: Study of samples S4 and S5. Pore (or trapped gas nanobubble) size and aspect ratio distribution histograms from TEM cross-sectional images, Figure S5: Study of samples S6 and S7. Pore (or trapped gas nanobubble) size and aspect ratio distribution histograms from TEM cross-sectional images, Figure S6: TEM cross section images of samples S1 and S4, Figure S7: SEM top-view images of samples S8 and S9.
