# Supplementary material for: Synergistic Effect of He for the Fabrication of Ne and Ar Gas-Charged Silicon Thin Films as Solid Targets for Spectroscopic Studies
Source: Nanomaterials (Basel). 2024 Apr 21;14(8):727. doi: 10.3390/nano14080727 (PMC11053770; doi:10.3390/nano14080727)
Supplement: Supplementary file 1 [file nanomaterials-14-00727-s001.zip › nanomaterials-2934730-supplementary.pdf]

SUPPORTING INFORMATION FILE

Synergistic effect of He for the fabrication of Ne and Ar gas-charged silicon films as solid targets for spectroscopic studies

A. Fernández*[1,], V. Godinho[1], J. Ávila[2], M.C. Jiménez de Haro[1], D. Hufschmidt[1], J. López-Viejobueno[1], G.E. Almanza-Vergara[1], F.J. Ferrer[3,4], J.L. Colaux[5], S. Lucas[5], M.C. Asensio[2,6]

[1] Instituto de Ciencia de Materiales de Sevilla (CSIC-Univ. Seville), Avda. Américo Vespucio 49, 41092-Sevilla, Spain.
[2] Synchrotron SOLEIL and Universite Paris-Saclay, L' Orme des Merisiers, BP48, 91190 Saint-Aubin, France.
[3] Centro Nacional de Aceleradores (Univ. Seville, J. Andalucía, CSIC), Av. Tomas Alva Edison 7, 41092, Seville, Spain.
[4] Departamento de FAMN (Univ. Seville), Aptd. 1065, 41012 Seville, Spain.
[5] Laboratoire d'Analyse par Réactions Nucléaires (LARN), Namur Institute of Structured Matter (NISM), University of Namur, 61 Rue de Bruxeles, 5000 Namur, Belgium.
[6] Madrid Institute of Materials Science (ICMM), CSIC, Cantoblanco, E-28049 Madrid, Spain.

* asuncion@icmse.csic.es

Fig. S1: Schematic drawing of the magnetron sputtering experimental set-up.

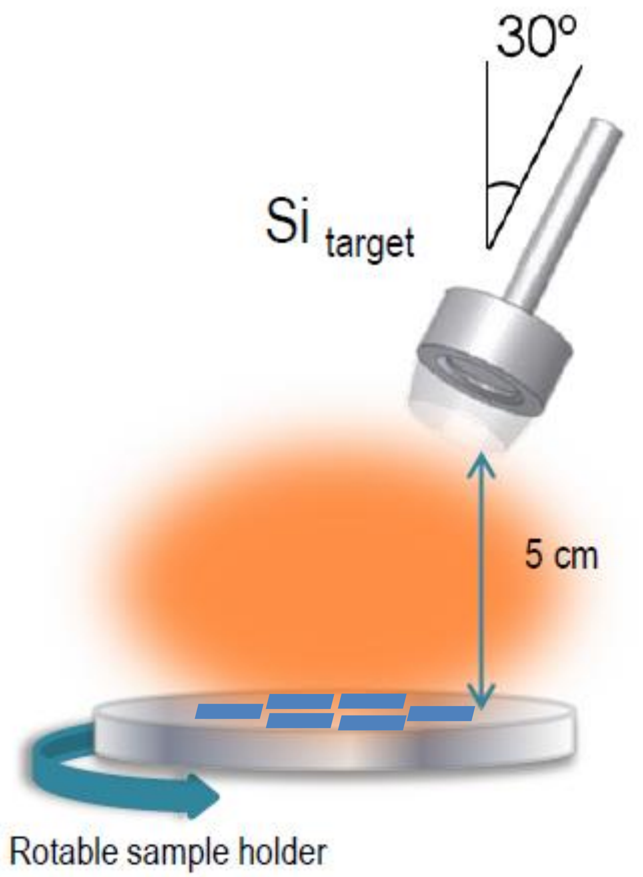

Fig. S2: Representative X-ray diffractrograms for samples S1, S3 and S4 deposited onto fussed quartz amorphous substrates. No characteristic diffraction peaks for crystalline Si were detected. The dashed line shows the position where the diffraction of the Si [111] planes should appear. The observed broad peak corresponds to the amorphous fussed quartz substrate.

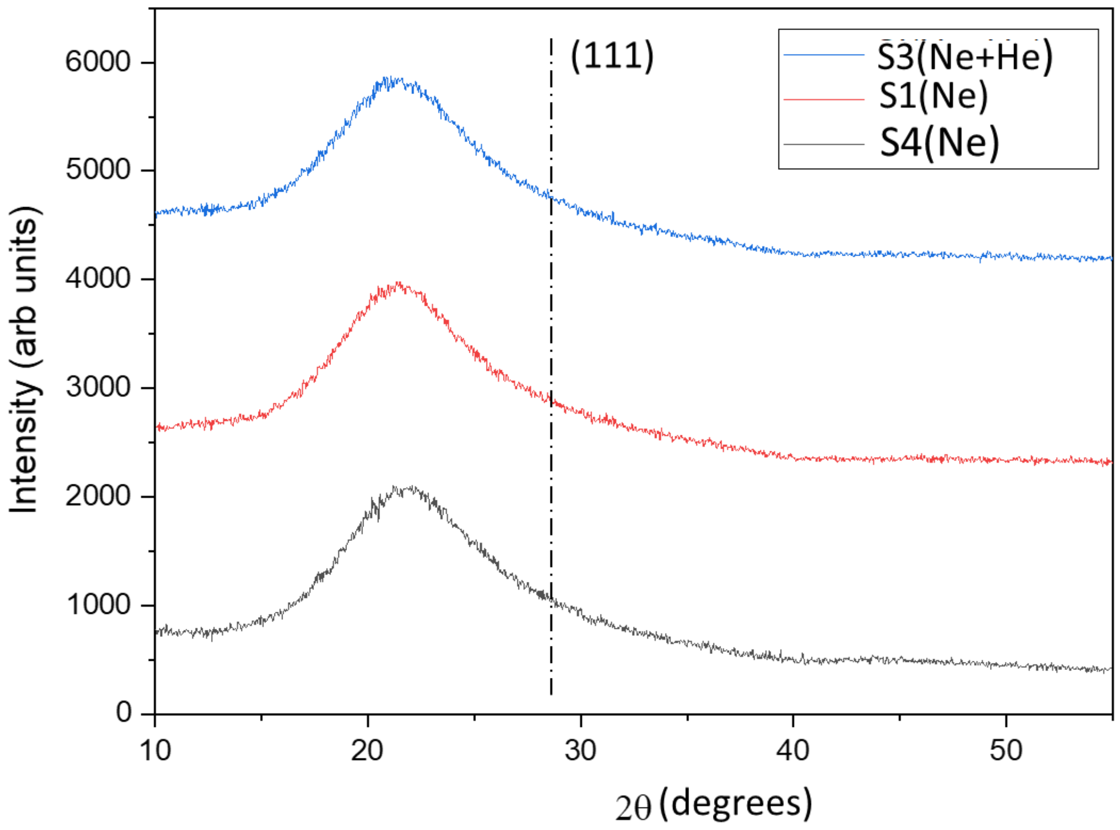

Fig. S3: Study of samples S1 and S2. Pore (or trapped gas nanobuble) size and aspect ratio distribution histograms from TEM cross-sectional images.

Sample S1:

Si-Ne/150dc/2Ne

Narower pore size distribution in the range 1-5 nm.
Pores are elongated with a mean aspect ratio of 0.6.

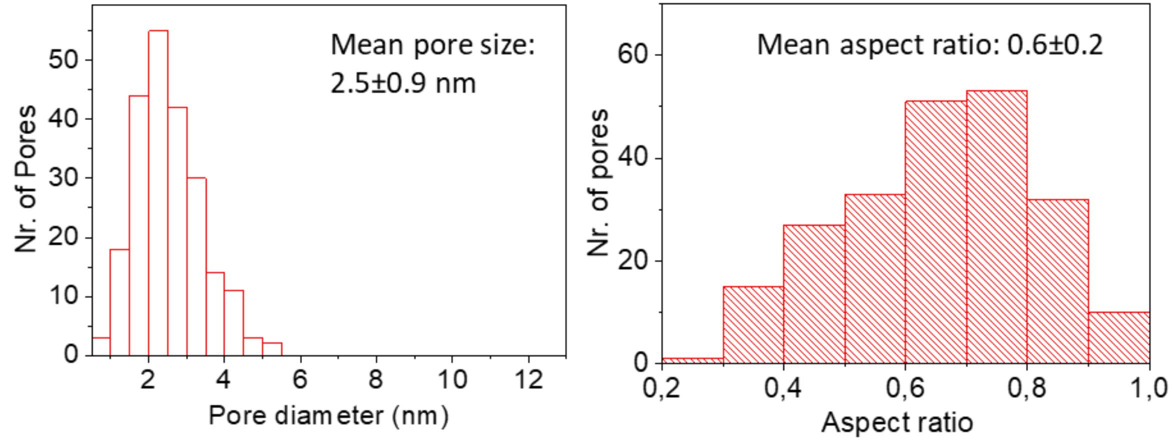

Sample S2:

Si-Ne(He)/150dc/2Ne+2He

Broader pore size distribution in the range 1-12 nm.
Pores are elongated with a mean aspect ratio of 0.6.

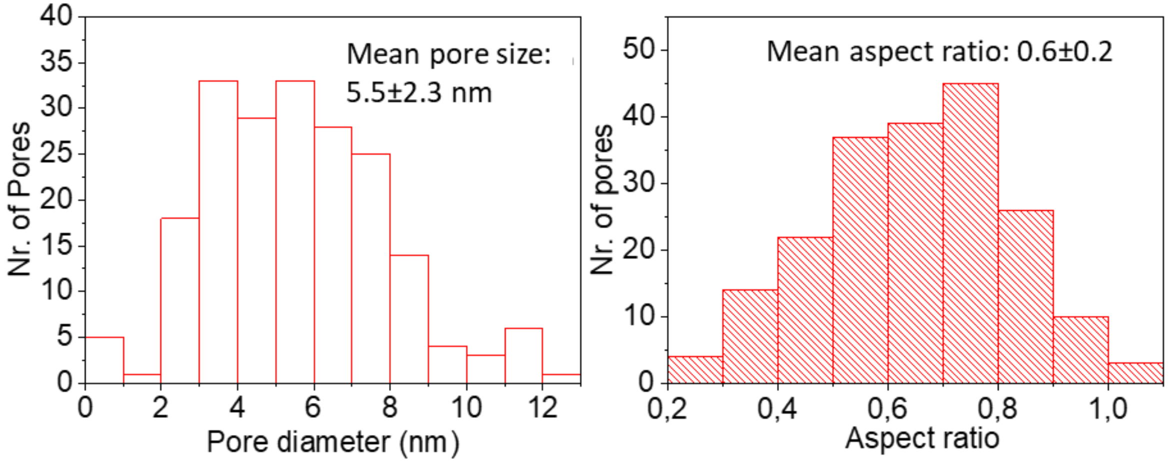

Due to the different pore shape (elongated) we have defined pore size as the diameter of a circle equivalent in area to the one obtained for each pore in the TEM images. Pores below 0.5 nm size are in the limit to what we could clearly identify in our images and we do not have considered them. The aspect ratio has been defined as minor to major axis ratio of the best fitted ellipse for each pore. A perfectly round pore has an aspect ratio of 1.0. Elongated pores have aspect ratios <1.0.

Fig. S4: Study of samples S4 and S5. Pore (or trapped gas nanobuble) size and aspect ratio distribution histograms from TEM cross-sectional images.

Sample S4:
Si-Ne/150rf/2Ne

Narower pore size distribution in the range 1-5 nm.
Pores are elongated with a mean aspect ratio of 0.6.

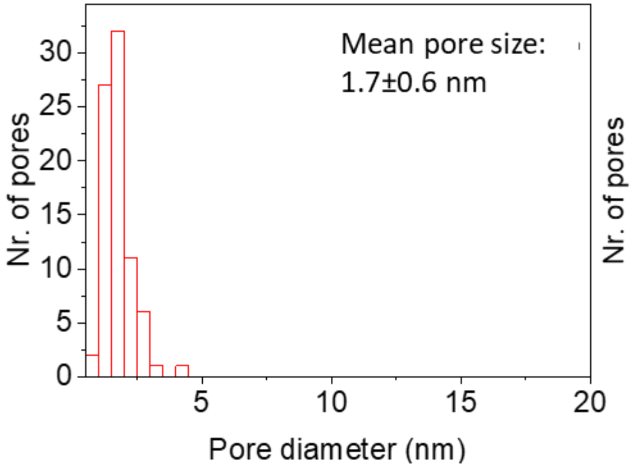

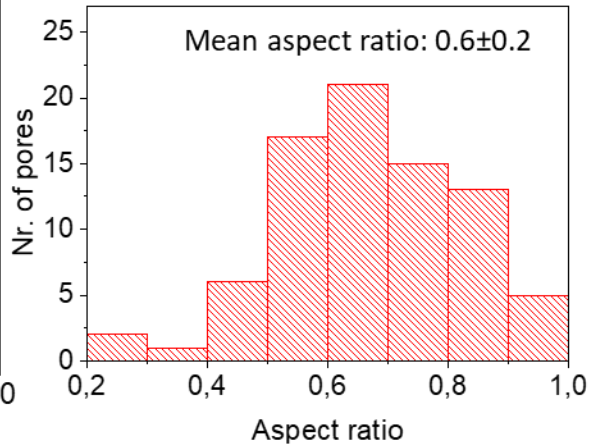

Sample S5:
Si-Ne(He)/150rf/2Ne+2He

Broader pore size distribution with some pores up to 20 nm.
Pores are elongated with a mean aspect ratio of 0.6.

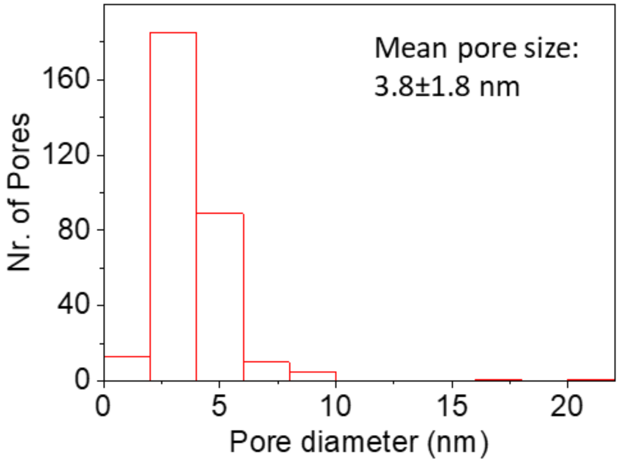

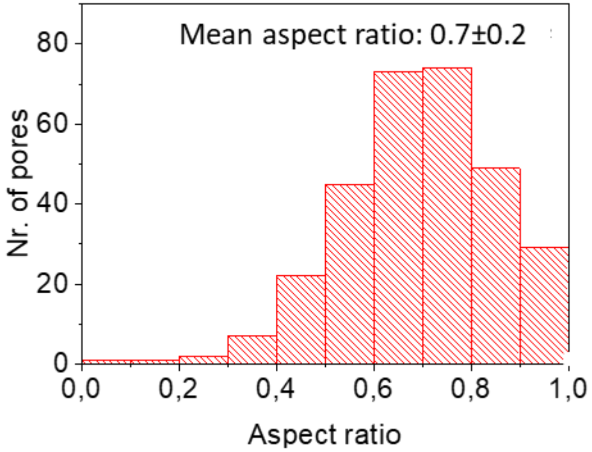

Due to the different pore shape (elongated) we have defined pore size as the diameter of a circle equivalent in area to the one obtained for each pore in the TEM images. Pores below 0.5 nm size are in the limit to what we could clearly identify in our images and we do not have considered them. The aspect ratio has been defined as minor to major axis ratio of the best fitted ellipse for each pore. A perfectly round pore has an aspect ratio of 1.0. Elongated pores have aspect ratios <1.0.

Fig. S5: Study of samples S6 and S7. Pore (or trapped gas nanobuble) size and aspect ratio distribution histograms from TEM cross-sectional images.

Sample S6:
Si-Ne/300dc/2Ne

Narower pore size distribution in the range 0.5-5 nm.
Pores are elongated with a mean aspect ratio of 0.7.

Sample S7:
Si-Ne(He)/300dc/2Ne+2He

Broader pore size distribution in the range 1-14 nm.
Pores are elongated with a mean aspect ratio of 0.6.

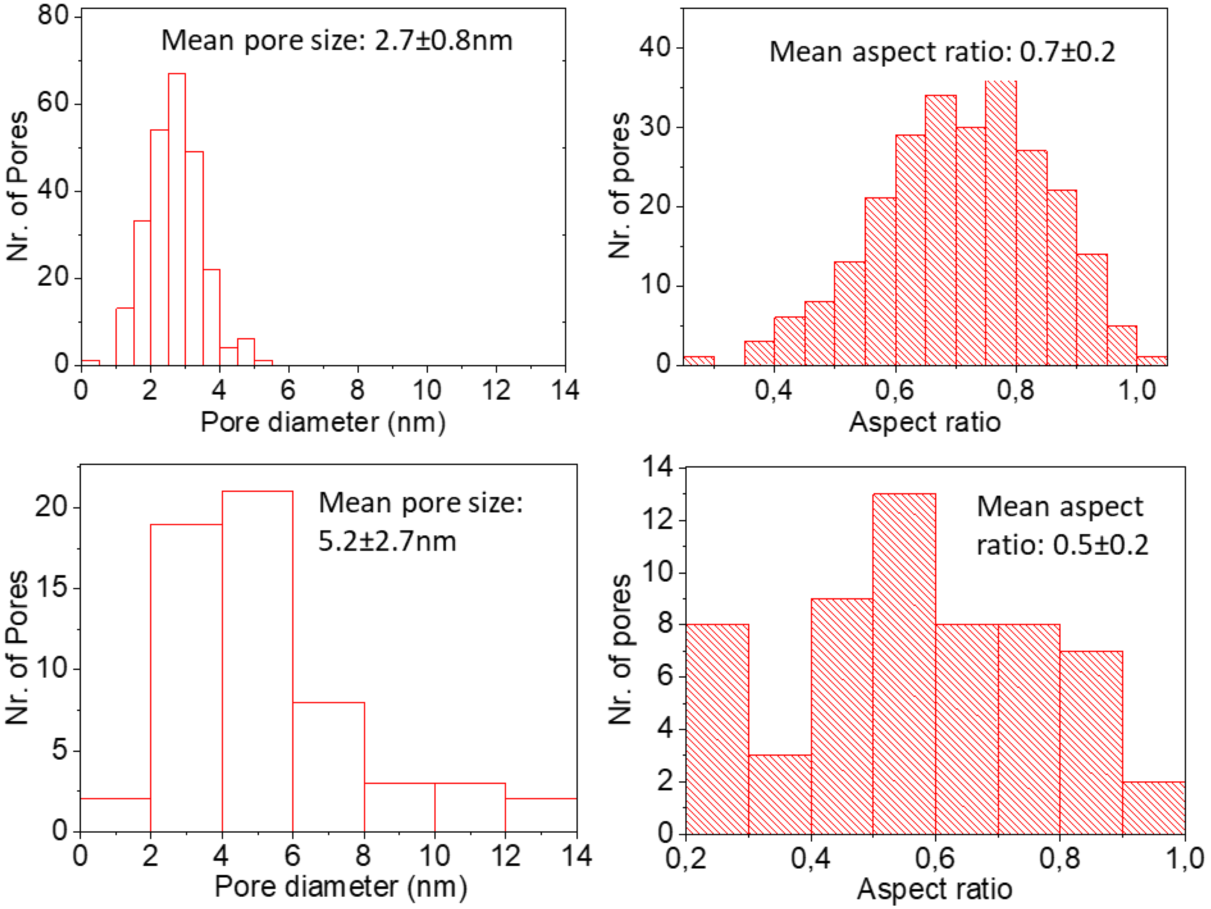

Due to the different pore shape (elongated) we have defined pore size as the diameter of a circle equivalent in area to the one obtained for each pore in the TEM images. Pores below 0.5 nm size are in the limit to what we could clearly identify in our images and we do not have considered them. The aspect ratio has been defined as minor to major axis ratio of the best fitted ellipse for each pore. A perfectly round pore has an aspect ratio of 1.0. Elongated pores have aspect ratios <1.0.

Fig. S6: TEM cross section images of samples S1 and S4. In these particular areas it was possible to visualize characteristic columns for films grown in pure Ne plasmas.

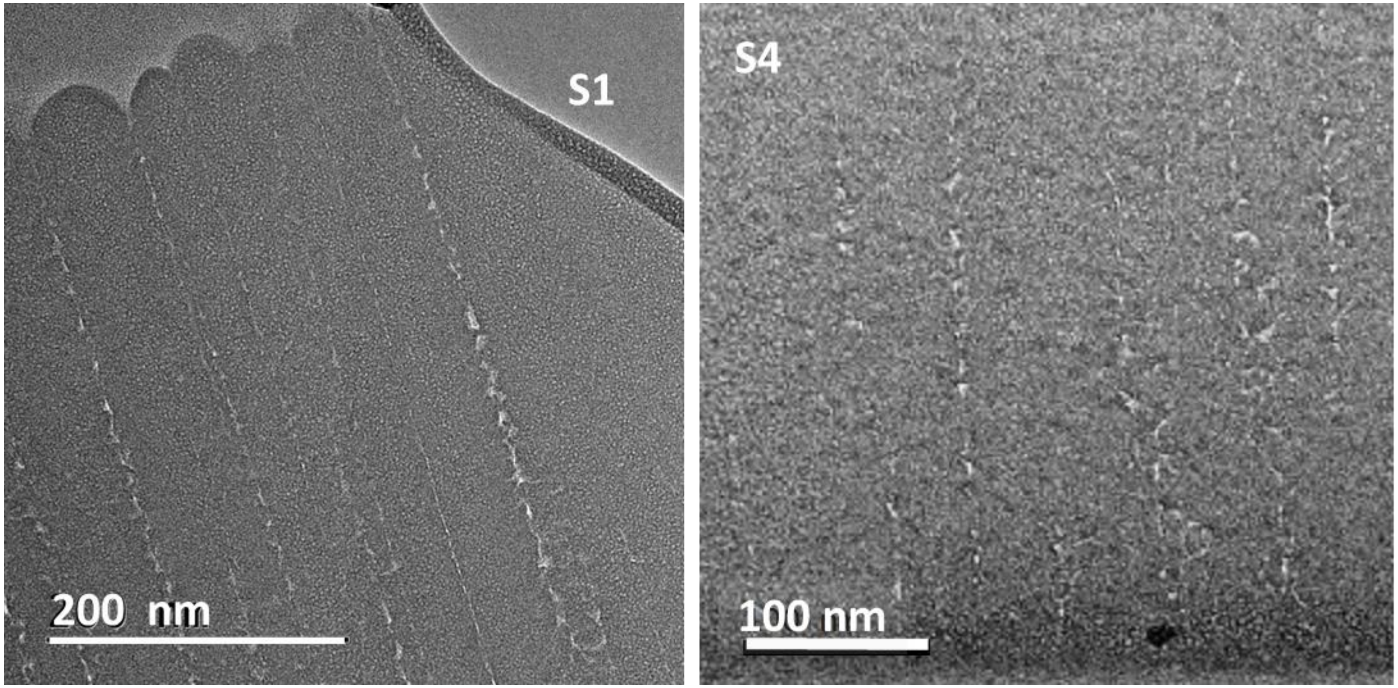

Fig. S7: SEM top-view images of samples S8 and S9. These images correspond to the observed columnar structures in the cross-section images.

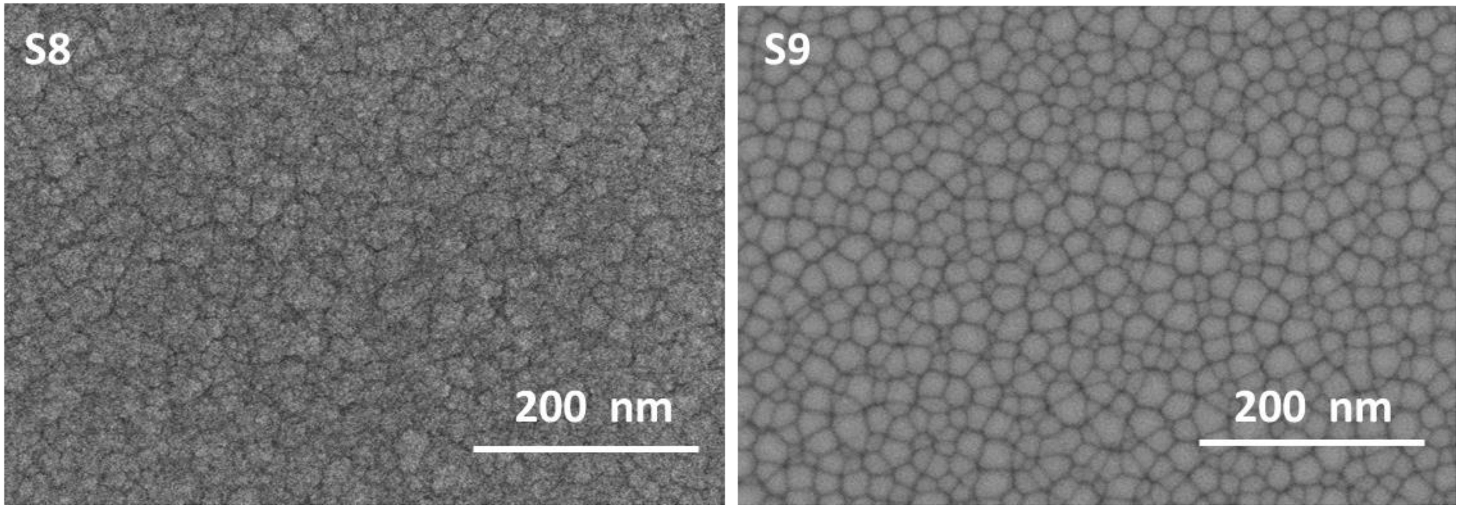

Sample nr.<br>Description	Column size range (nm)	Mean column size (nm)
**S8:**<br>Si-Ar/150dc/2Ar	14-89	39±23
**S9:**<br>Si-Ar(He)/150dc/1Ar+1He	17-85	49±26
